# Supplementary figures and images for: In-silico and in-vitro morphometric analysis of intestinal organoids
Source: PLoS Comput Biol. 2023 Aug 14;19(8):e1011386. doi: 10.1371/journal.pcbi.1011386 (PMC10473498; doi:10.1371/journal.pcbi.1011386)

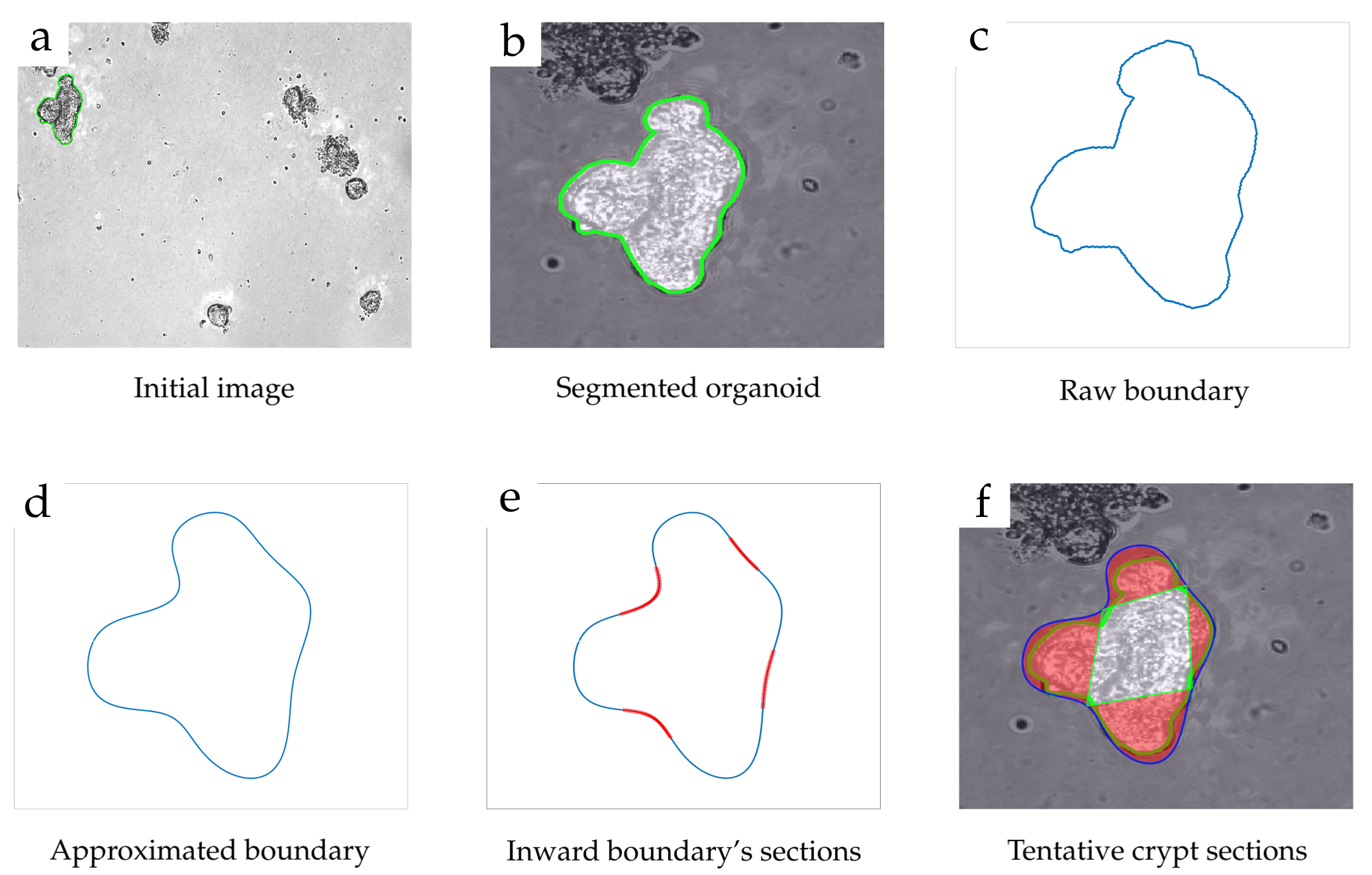

Supplement: S1 Fig — This image shows the general processing performed for each organoid’s boundary to calculate the number of crypts present after extracting the mask’s information. (a) Initial stacked image of a 3-day-old organoid culture; (b) manual segmentation of an organoid; (c) raw boundary extracted from the segmented organoid; (d) boundary approximation obtained using a Fourier approximation; (e) calculated concave sections (in red) on the boundary; and (f) possible crypt sections detected by our algorithm. (TIFF) [file pcbi.1011386.s001.tiff]

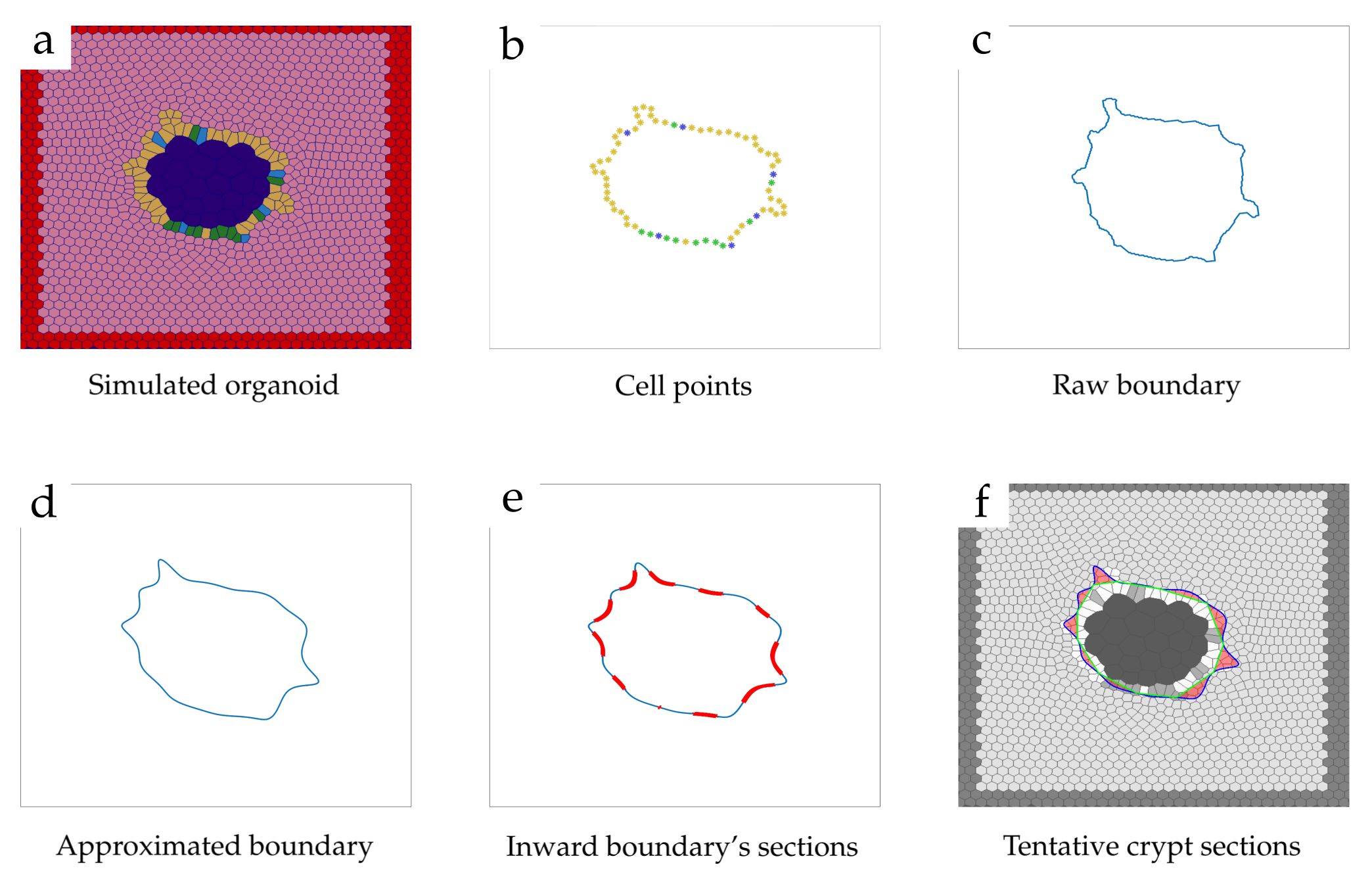

Supplement: S2 Fig — This image shows the general processing performed for each in-silico organoid’s boundary to calculate the number of crypts presented after extracting the mask’s information. (a) Initial image obtained from a 3 day old organoid simulation with several nodes representing stem cells (blue), transit amplifying cells (yellow), Paneth cells (green), Matrigel (pink), lumen (dark blue) and the simulation boundary (red); (b) location of epithelial cells (SC (blue), TA (yellow), PC (green)) extracted from the simulation; (c) raw boundary of the simulated organoid; (d) boundary approximation obtained using a Fourier approximation; (e) calculated concave sections (in red) on the boundary; and (f) possible crypt sections detected by our algorithm. (TIFF) [file pcbi.1011386.s002.tiff]

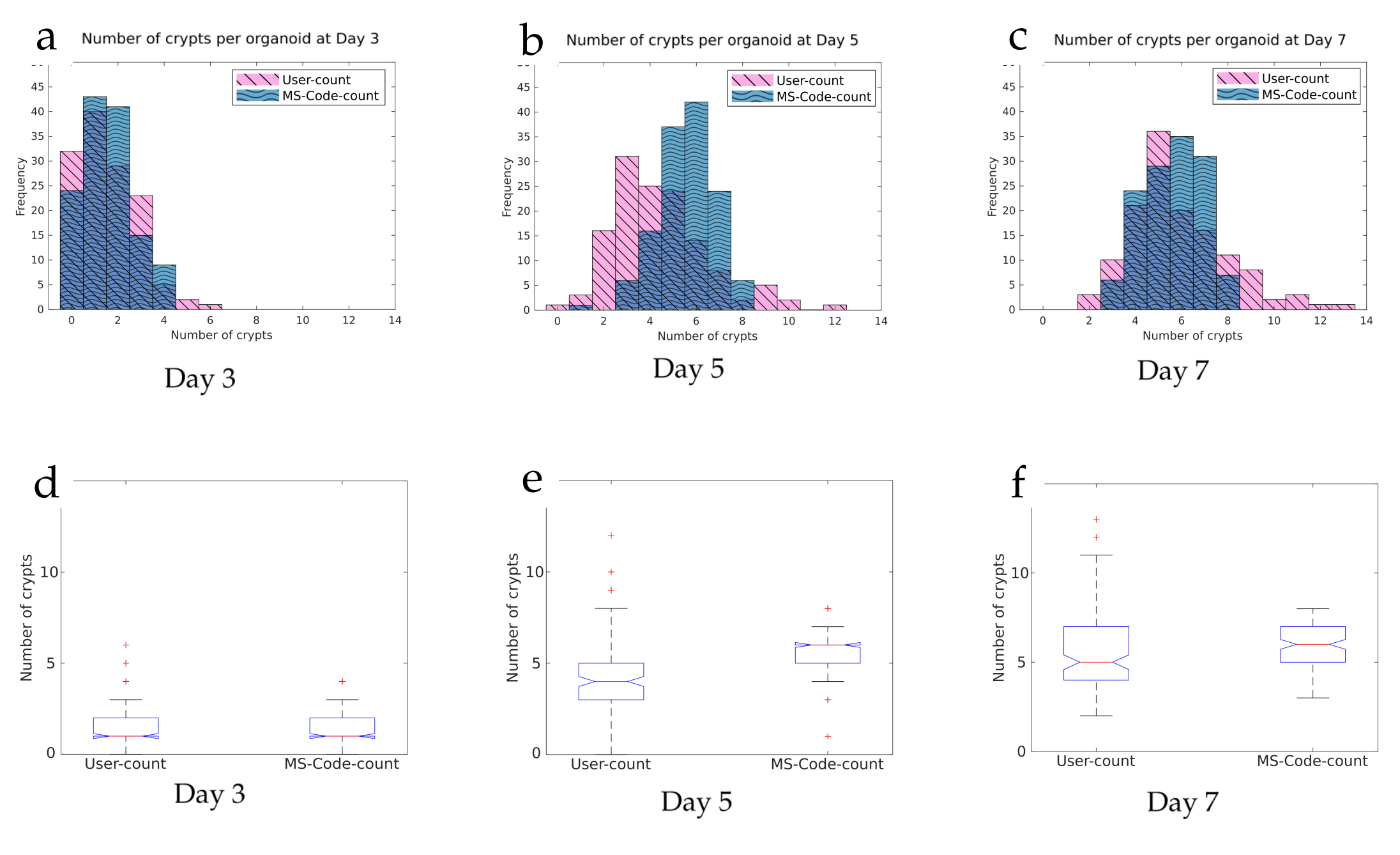

Supplement: S3 Fig — Comparison of number of crypts found per organoid at each day by the user in comparison to those found in ‘MS-Code-count’. (a-c) Histograms of the distribution of crypts found by the user (pink), compared to ‘MS-Code-count’ (blue). (d-f) Boxplots of the number of crypts found using the previously mentioned methods, in which the boundaries of the box represent the 25th and 75th percentiles respectively of the median (red line) number of crypts found per day, the width of the notch represents a 95% confidence interval around the median, the whiskers extend the most extreme data points, and the outliers are represented individually (red plus signs). (TIFF) [file pcbi.1011386.s003.tiff]

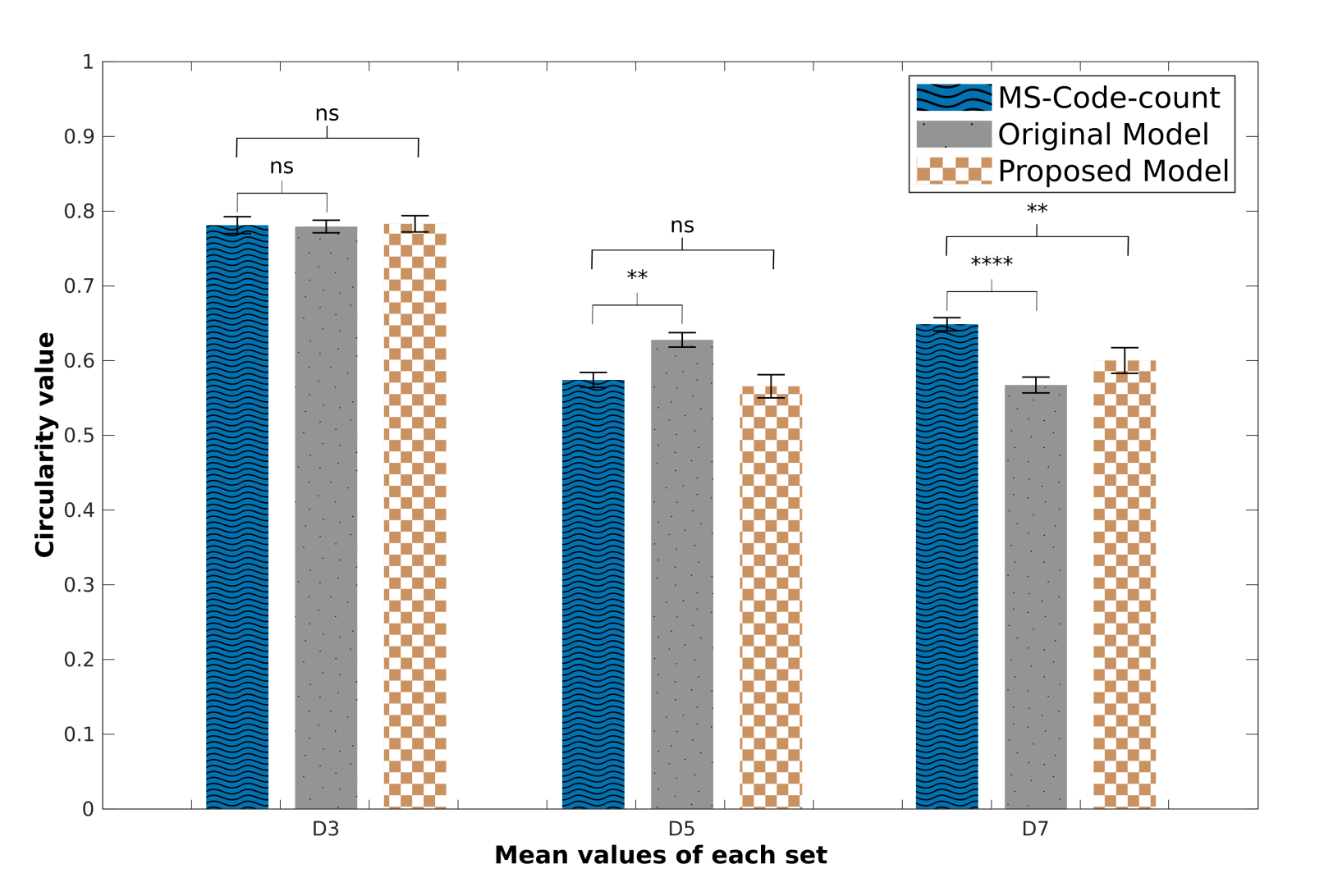

Supplement: S6 Fig — Comparison of mean circularity calculated at each day for in-vitro (using the manually segmented images) and in-silico organoids. P-values from two-tailed unpaired t-test computed over the data sets shown, *p<0.05, **p<0.01, ***p<0.001, ****p<0.0001. (TIFF) [file pcbi.1011386.s006.tiff]
